# Buckling stability analysis of underpinning piles during basement excavation beneath existing buildings

**DOI:** 10.1038/s41598-022-11791-8

**Published:** 2022-05-17

**Authors:** Huafeng Shan, Jie Yang, Zhiwen Hu, Shaoheng He

**Affiliations:** 1 School of Civil Engineering and Architecture, Taizhou University, Taizhou, 318000 China; 2 Zhejiang Fangyuan New Materials Co., Ltd., Taizhou, 318000 Zhejiang China; 3 Taizhou Huangyan Municipal Garden Construction and Development Co., LTD., Taizhou, 318000 Zhejiang China; 4 Research Center of Costal and Urban Geotechnical Engineering, Zhejiang University, Hangzhou, 310058 Zhejiang China

**Keywords:** Civil engineering, Geophysics

## Abstract

In the present paper, the pile-soil system’s total potential energy equation of underpinning piles was established based on the Winkler elastic foundation beam theory. This energy equation was used to explore the effect of basement excavation beneath existing buildings on the underpinning pile’ buckling stability. Utilizing the minimum potential energy theory, the expression of the critical buckling load for underpinning piles’ stability during the excavation project was obtained. Moreover, the influencing factors of the critical buckling load were investigated. It was found that the underpinning pile’s critical load converged with the augment of half-wave number. Moreover, the pile skin friction and deadweight had an insignificant influence on it. In addition, the critical load of underpinning piles decreased sharply with the increasing excavation depth and gradually increased with the augment of pile diameter. The results of this study provides a basis for the design of adding piles in similar projects and reduces the hidden danger of excavation instability.

## Introduction

The technology of basement excavation beneath existing buildings is to underpin the foundation for existing buildings without damaging the superstructure, and then the earthwork is excavated to the design depth and the basement foundation slab is poured to add a basement. This technology has attracted extensive attention locally and globally because it can not only solve the problem of insufficient underground space for the old buildings downtown, giving these buildings a “second life”, but also avoid the economic loss caused by the demolition and reconstruction.

At present, some scholars have done considerable research on this issue. Iwasaki et al.^1^ introduced a new subway tunnel project of an underground shopping mall in Nagoya, Japan. Jia et al.^2^ using the finite element method, simulated the working conditions of basement excavation beneath a three-storey frame structure. Gong et al.^3^ researched the lateral friction mechanism of existing piles during basement excavation beneath existing buildings. Wu et al.^4,5^ devised an approach to explain the mechanism of pile end resistance and analyzed the impact of excavation construction on the existing piles’ bearing stiffness. Shan et al.^6^ extended this method to pile group foundations of existing buildings and studied the impact of excavation on settlement characteristics of existing pile group foundations. Note that all the above analyses focus on the impact of basement excavation beneath existing buildings on the bearing capacity and settlement of the original foundation piles. Wang et al^7–9^ studied the deformation and internal force of pile.

As for the buckling stability of pile foundations, early studies illustrate that buckling instability will not occur when piles of the ordinary size are pressed into soft soil. However, with long piles and super-long piles, buckling problems of foundation piles will arise^10^. For instance, Reddy et al.^11^ experimentally studied the buckling instability phenomenon of foundation piles through laboratory tests. Lee et al.^12^ put forward an energy approach for calculating the buckling and instability of foundation piles. Using the Ritz method, Zhu^13^ obtained the calculation length for stability of bridge pile foundations. Zhao^14,15^ proposed different buckling load calculation expressions under different constraint conditions of foundation piles. Zou et al.^16^, using the energy method, studied the buckling stability of rock-socketed piles with a highrise pile cap under the complex subgrade reaction modulus.

However, the effect of excavation on the foundation piles’ stability has not been studied yet. During basement excavation beneath existing buildings, with the increasing excavation depth, the soil layer constraint around the pile on the foundation pile gradually disappears, resulting in the increase of the foundation pile’s free length, which is likely to cause buckling and instability of foundation piles under the pile top load. Therefore, it is necessary to study the influence of excavation on the foundation pile’s buckling stability. Herein, based on the basement construction project of Ganshuixiang in No. 3 section (hereafter called the Ganshuixiang project), combined with the Winkler elastic foundation beam theory, the pile-soil system’s total potential energy equation was established. By using the minimum potential energy theory, the expression of the critical buckling load and calculation length for underpinning piles’ stability was obtained. The influences of half-wave number, pile deadweight, pile skin friction, pile diameter and excavation depth on the underpinning piles’ critical buckling load were analyzed, which could provide a reference for relevant projects.

## Project overview

**Figure 1:**
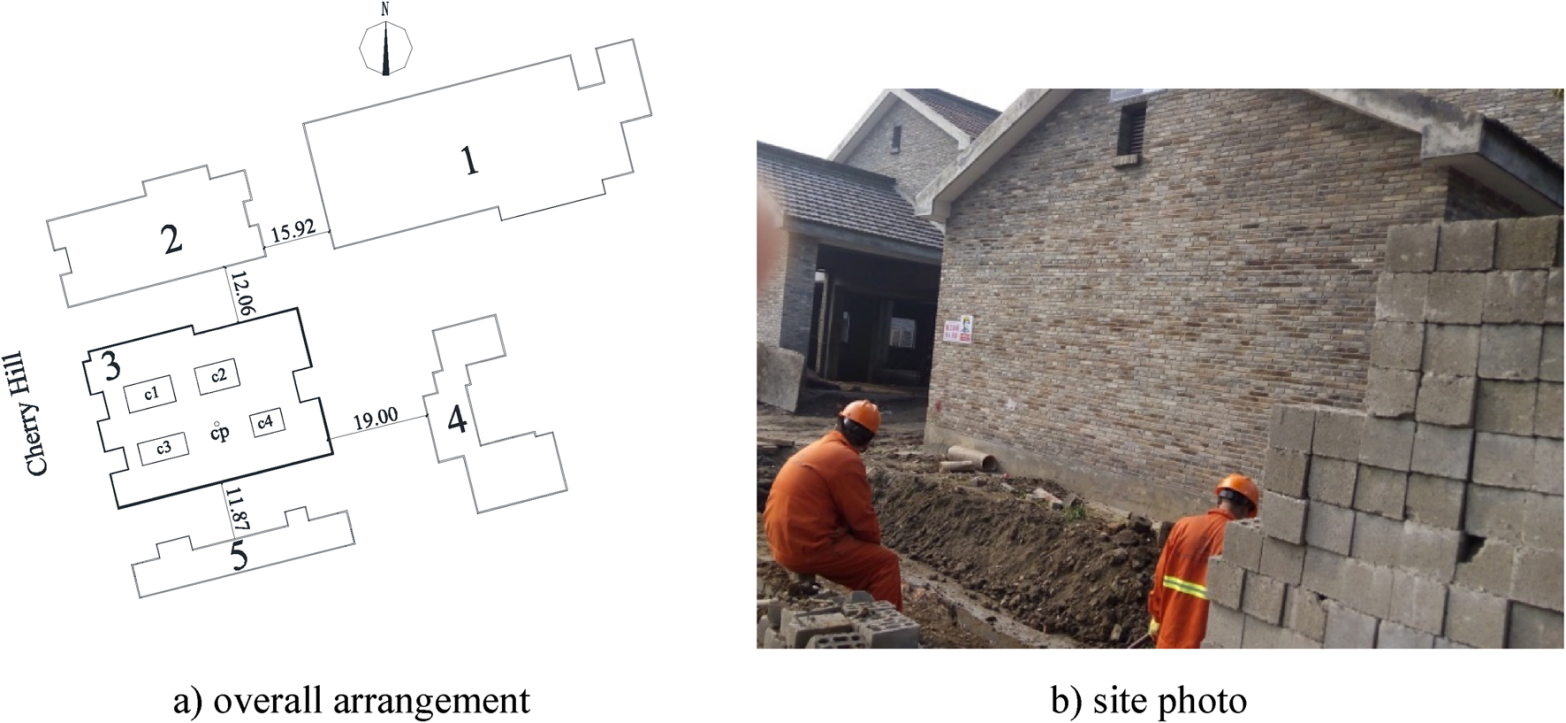
Overall arrangement and site photo of the Ganshuixiang project (unit: m). 1–5 denote No. 1–5 section of Ganshuixiang; c1-4 denote No. 1–4 courtyard; cp is soil sampling point;

The Ganshuixiang project lies in Ganshuixiang, Zhakou Street, Shangcheng District, which is a typical project in Hangzhou, Zhejiang Province. This project is proximate to the White Pagoda Park. To keep harmony with the environment of the scenic spot, this project adopts an antique building with a building height of 8.01 m. The main body is a frame structure with two stories (partially one-story), and without basement. No. 2 section and No. 5 section of Ganshuixiang are located in the north and south of this project. The building in the east direction is No. 4 section which is under construction, and Cherry Hill lies in the west. The detailed layout is displayed in Fig. 1. The Ganshuixiang project is a case in typical soft soil. Its physico-chemical properties are given in Table 1. As the superstructure load is tiny, an isolated foundation under the pillar with a 1.80-m buried depth is utilized.

**Table 1:** Physico-chemical properties of soils.

Layer no	Name	Layer thickness (m)	Soil unit weight (kN/m^3^)	Cohesion (kPa)	Internal friction angle (°)	Compression modulus (MPa)	Poisson’s ratio	Friction eigenvalue (kPa)
1	Miscellaneous fill	0.9	–	–	–	–	–	–
2	Silty clay	0.6	18.70	8.9	3.6	3.5	0.35	–
3	Clay silt	6.1	18.55	12.1	27.4	10.5	0.35	20
4	Sludge	3.8	16.15	8.5	2.9	2.5	0.35	7
5	Gravelly silty clay	1.9	19.00	40.0	13.8	6.0	0.35	25
6	Fully weathered sandstone	2.5	19.77			15.0	0.35	
7	Highly weathered sandstone	2.2	19.77	146.3	20.5	25.0	0.25	55
8	Moderately weathered sandstone	–	–	–	–	> 50.0	0.25	75

After the completion of the project, the community owners discovered that the building lacked sufficient usable area. To this end, they planned to add a basement beneath the existing building with an estimated excavation depth of 4.42 m.

## Construction technology of basement excavation

**Figure 2:**
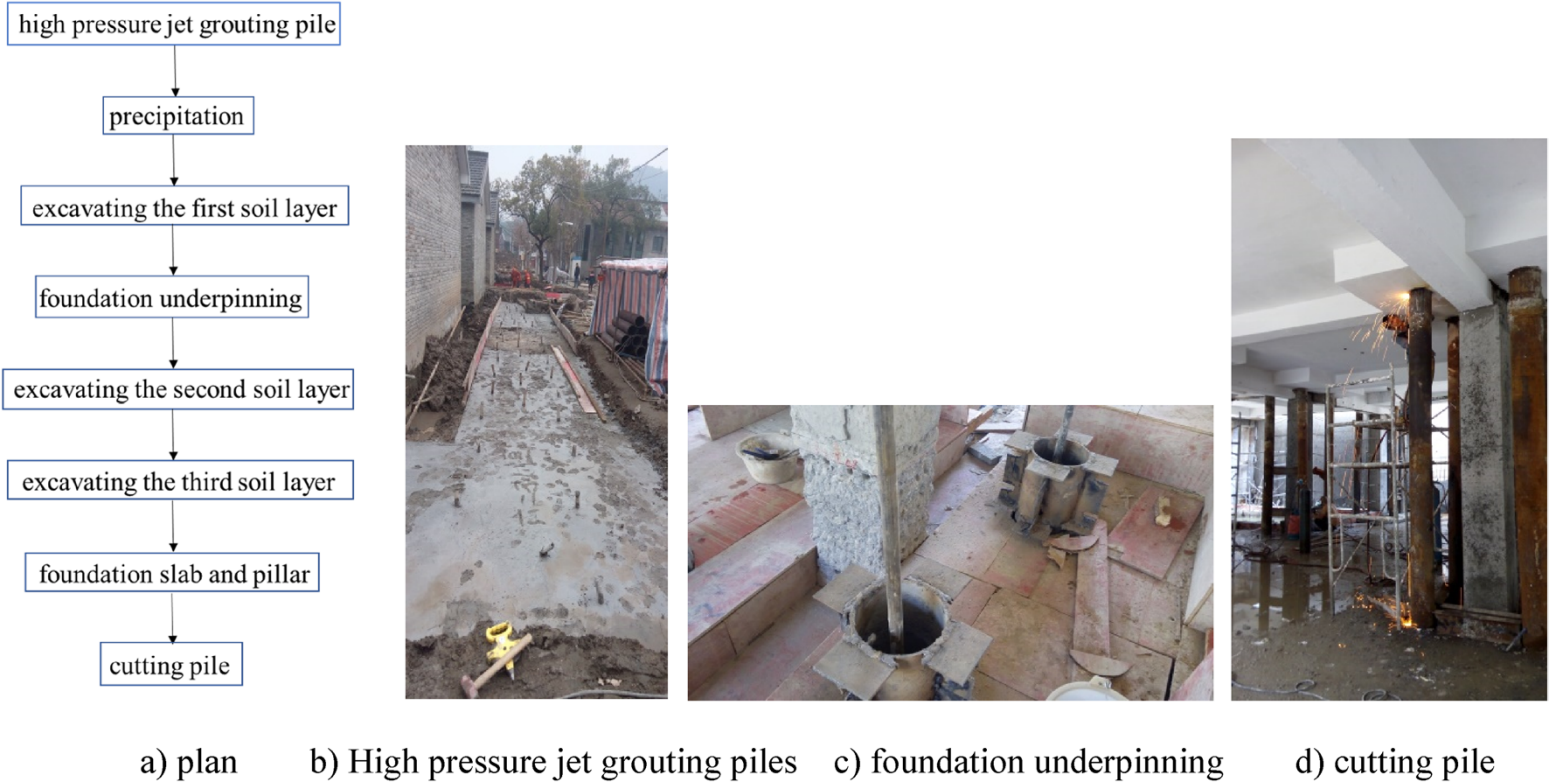
Construction flowchart of the Ganshuixiang project.

**Figure 3:**
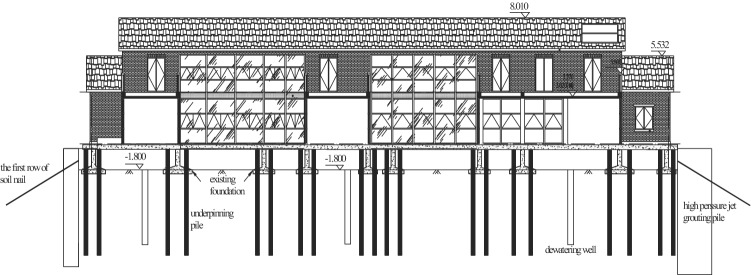
Excavating the first soil layer and constructing underpinning piles (unit: m).

Different superstructures and foundations need different excavation plans^17^. This section introduced the construction flow of the project, as illustrated in Fig. 2:

The detailed technology for construction is as follows:

1. High pressure jet grouting piles were built as the building envelope of the foundation pit, as shown in Fig. 2b.
2. The first soil layer had an excavation depth of 1.80 m, i.e., the buried depth of the isolated foundation. Then, the first layer of the soil nailing wall was built, as given in Fig. 3.
3. The second soil layer had an excavation depth of 3.0 m. With the deeper excavation depth, the soil constraint on the pile side gradually dropped. Subjected to the pile top’s axial load, the foundation pile might buckle and become unstable. Therefore, when the excavation depth came up to 3.0 m, steel supports should be welded between foundation piles, as presented in Fig. 3, to avoid buckling instability of these piles. Then, the second layer of the soil nailing wall was built.
4. The third soil layer had an excavation depth of 4.42 m, and the third layer of the soil nailing wall was built.
5. Subsequently, the basement cushion as well as the foundation slab was poured. Then, the initial isolated foundation and foundation beam were chiselled away. The new basement structural pillar was poured.
6. To improve the new basement’s space use rate, underpinning piles ought to be removed in this project, as shown in Fig. 2d.

The Ganshuixiang project’s main structure was completed at this point.

## Buckling stability equation

### Buckling analysis

**Figure 4:**
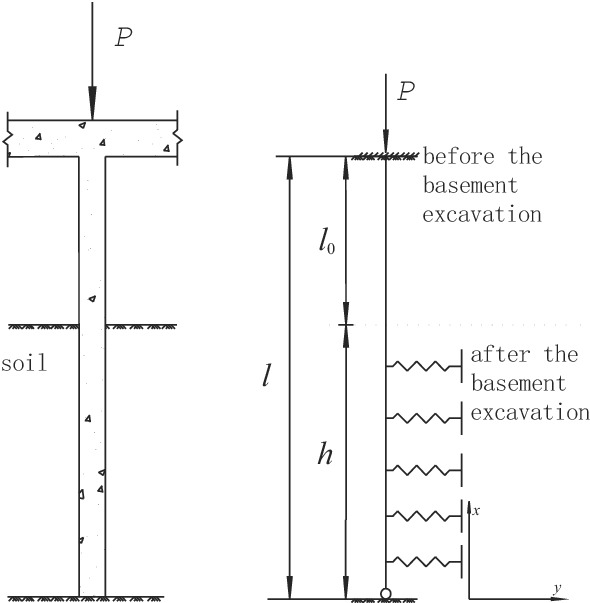
Buckling analysis model.

In practical engineering cases, it is difficult to achieve ideal consolidation between pile and cap, and the pile top constraint is usually between fixed support and hinged support. Therefore, this paper assumed that pile top constraint was elastic embedding. However, in this project, steel pipe piles were not rock-socketed. They only stood on stable rock strata. Therefore, this paper assumed that the pile end constraint was hinged support. Due to the complicated pile-pile and pile-soil interactions in pile group foundations, this paper only analyzed the effect of excavation beneath existing buildings on the single pile foundation’s buckling instability, and the excavation method of soil layer was considered as layered excavation. Its calculation model is shown in Fig. 4.

The interaction between the soil layer around the pile and the pile was simulated by Winkler springs. Therefore, the subgrade reaction *q*(x) could be obtained by the *m* method recommended by the specification^18^:

1 $$ q(x) = kyb_{0} = m(h - x)yb_{0} \begin{array}{*{20}l} {} & {(0 \le x \le h)} \\ \end{array} $$

where *k* is the subgrade reaction coefficient; *b*_0_ is the calculation width; *m* is the coefficient.

Since the horizontal load test of underpinning piles had not been carried out in this project, the following empirical formula was adopted to calculate the *m* value^18^.

2 $$ m = \frac{{0.2\varphi^{2} - \varphi + c}}{{v_{b} }} $$

where *φ* is soil layer’s friction angle; *c* is its cohesion; *v*_*b*_ is the horizontal displacement value, taken as 10 mm in the soft soil area^19,20^.

Since the site of this project was multi-layer soil rather than homogeneous soil layer, this paper took the weighted average of soil layer thickness as suggested by Chen et al.^21^.

According to the existing test data^22^, the calculation width could be expressed as:

3 $$ b_{0} = k_{f} k_{0} kd $$

where *k*_*f*_ is the character conversion coefficient; *k*_0_ is the force conversion coefficient; *k* is the coefficient of mutual influence between piles; *d* is the pile diameter.

The calculation of Eq. (3) is complex and its parameters are difficult to determine. Therefore, a simplified calculation method is proposed in China^20,22^.

In terms of circular piles:

4 $$ \left\{ {\begin{array}{*{20}l} {b_{0} = 0.9(1.5d + 0.5) \quad d \le 1\, m} \\ {b_{0} = 0.9(d + 1) \quad d \le 1\, m} \\ \end{array} } \right. $$

For rectangular piles:

5 $$ \left\{ {\begin{array}{ll} {b_{0} = 1.5d + 0.5\quad d \le 1m} \\ {b_{0} = d + 1\quad d > 1m} \\ \end{array} } \right. $$

Combined with the restraint conditions of pile top and pile end, according to the Ritz method, the buckling deformation of the pile was as follows:

6 $$ y = \sum\limits_{i = 1}^{n} {c_{i} \sin \frac{2i - 1}{{2l}}} \pi x $$

where *c*_*i*_ is the undetermined coefficient; *l* is the length of the pile; *n* is the half-wave number.

### Establishing the pile buckling equation

The pile-soil system’s total potential energy (*Π*) is composed of soil’s elastic deformation energy around the pile (*U*_*s*_), pile’s bending strain energy (*U*_*p*_), pile top’s load potential energy (*V*_*p*_), pile’s deadweight potential energy (*V*_*g*_) and pile skin friction-induced load potential energy (*V*_*f*_), namely:

7 $$ \Pi = U_{p} + U_{s} + V_{p} + V_{f} + V_{g} $$

The soil’s elastic deformation energy around the pile *U*_s_:

8 $$ U_{s} = \frac{1}{2}\int_{0}^{h} {q(x)ydx} = \frac{{mb_{0} }}{2}\int_{0}^{h} {(h - x)y^{2} } dx $$

The pile’s bending strain energy *U*_*p*_:

9 $$ U_{p} = \frac{EI}{2}\int_{0}^{l} {\left( {y^{^{\prime\prime}} } \right)}^{2} dx $$

where *E* is the pile’s elastic modulus, which is 2.06×10^5^ MPa; *I* is the pile second moment of area, *I*=*π*×(*D*^4 ^− *d*^4^)/64, where *D* and *d* are the outer diameter and inner diameter of the steel pipe plie, respectively.

**Figure 5:**
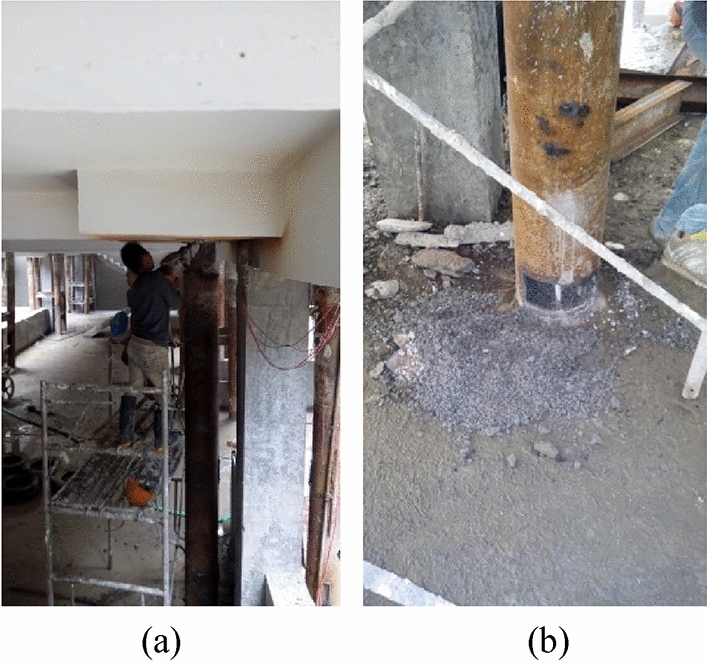
Fine aggregate concrete at the pile top and pile end in the field: (**a**) Pile top; (**b**) Pile end.

The underpinning piles were began to cut on June 23, 2015. It can be seen from the cutting position that the fine aggregate concrete at the pile top had good condensation and high strength, so it needed to be chiseled away with a pneumatic pick, as shown in Fig. 5a. However, the fine aggregate concrete at the pile end had poor condensation and was fragmentary, as presented in Fig. 5b. The explanation may be that the ground water level of the project site is high, which has a certain influence on the solidification of fine aggregate concrete. Since the strength of fine aggregate concrete was relatively discrete, its strength effect was not considered in this paper.

The load potential energy at the pile top *V*_*p*_:

10 $$ V_{p} = - \frac{P}{2}\int_{0}^{l} {\left( {y^{\prime}} \right)}^{2} dx $$

In this paper, the pile deadweight was simplified as uniform line load, then the deadweight potential energy of the pile (*V*_*g*_) was:

11 $$ V_{g} = - \frac{\gamma A}{2}\int_{0}^{l} {(h - x)\left( {y^{\prime}} \right)}^{2} dx $$

where *γ* is the unit weight of the foundation pile; *A* is the pile’s sectional area.

Since the mechanism of pile skin friction was complex and difficult to determine, this paper assumed the uniform distribution of pile skin friction to simplify the calculation. Then, the load potential energy caused by pile skin friction (*V*_*f*_) was:

12 $$ V_{f} = \frac{U\tau }{2}\int_{0}^{h} {(h - x)\left( {y^{\prime}} \right)}^{2} dx $$

where *U* is the perimeter of the pile section; *τ* is the skin friction on the pile.

Substituting Eqs. (8)–(12) into Eq. (7), the equation for *Π* of the pile-soil system was:

13 $$ \begin{aligned} \Pi & = \frac{EI}{2}\int_{0}^{l} {\left( {y^{^{\prime\prime}} } \right)}^{2} dx + \frac{{mb_{0} }}{2}\int_{0}^{h} {(h - x)y^{2} } dx  \hfill \\ & \quad + \begin{array}{*{20}c} {} & {} \\ \end{array} \frac{U\tau }{2}\int_{0}^{h} {(h - x)\left( {y^{\prime}} \right)}^{2} dx - \frac{P}{2}\int_{0}^{l} {\left( {y^{\prime}} \right)}^{2} dx_{p} \hfill \\ \begin{array}{*{20}c} {} & {} \\ \end{array} & \quad - \frac{\gamma A}{2}\int_{0}^{l} {(h - x)\left( {y^{\prime}} \right)}^{2} dx \hfill \\ \end{aligned} $$

Based on the minimum potential energy theory^23^, the following equation could be obtained:

14 $$ \frac{\partial \Pi }{{\partial c_{i} }}{ = 0}\begin{array}{*{20}l} {} & {i = 1,2,3} \\ \end{array} \cdots n $$

Then, the foundation pile’s buckling stability equation was *D* = 0, and its form was as follows:

15 $$ D = \left| {\begin{array}{*{20}c} {a_{11} - x} & {a_{12} } & \cdots & {a_{1n} } \\ {a_{21} } & {a_{22} - x} & \cdots & {a_{2n} } \\ \vdots & \vdots & \vdots & \vdots \\ {a_{n1} } & {a_{n2} } & \cdots & {a_{nn} - x} \\ \end{array} } \right| = 0 $$

where *x* = *Pl*^2^/(π^2^EI) ; *a*_ij_ is an element of the determinant *D*, which is related to pile length, embedded depth, pile diameter and pile skin friction.

Note that *n* eigenvalues of the above determinants could be obtained by the Jacobian determinant. The minimum eigenvalue was taken as *x*_*min*_, then the pile’s critical buckling load (*P*_*cr*_) was:

16 $$ P_{cr} = \frac{{\pi^{2} EI}}{{l^{2} }}x_{\min } $$

## Parametric analysis

The underpinning pile’s ultimate bearing capacity in this project was 900 kN, so the load eigenvalue of the underpinning piles was 450 kN. Therefore, in the following analysis, the load on the pile top was assumed to be 450 kN, and the steel pipe pile (outer diameter: 250 mm; wall thickness: 8 mm) was adopted for analysis.

### Influence of half-wave number

**Figure 6:**
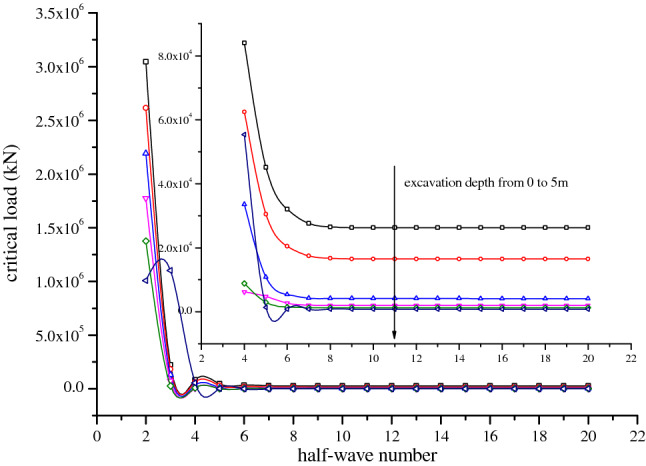
Influence of half-wave number on the critical load at different excavation depths.

As shown in Fig. 6, influenced by soil resistance at the pile end, the value of the function half-wave number was extremely sensitive to the calculation accuracy of the critical load. As can be seen from the figure, at the same excavation depth, the function half-wave number *n* increased from 2 to 3, and the corresponding critical load decreased sharply. Afterwards, the critical load slowly converged with the increase of half-wave number. When the half-wave number *n* = 15, the critical load at different excavation depths converged, which was basically consistent with the conclusion proposed by Zhao^14,15^ that when the half-wave number *n* ≥ 16, the critical load of foundation piles converged. Therefore, the half-wave number used in the following analysis was 15.

### Influences of skin friction and pile deadweight

Under the vertical load, the pile skin friction is a key parameter. This section analyzed the influences of pile skin friction and pile deadweight on the critical buckling load under the same excavation depth, as shown in Table 2, where *α*_*w*_, *α*_*s*_, and *α*_*ws*_ are the ratio between the critical buckling load of foundation piles without considering the pile deadweight, pile skin friction, and both at the same excavation depth, and that considering both, respectively. As presented in Table 2, at the same excavation depth, the *α*_*w*_, *α*_*s*_, and *α*_*ws*_ values were all close to 1.0. Therefore, the influences of skin friction and pile deadweight on the critical buckling load were negligible, which was consistent with the reports in the literature^14,24^.

**Table 2:** Influences of skin friction and pile deadweight on the critical load.

Excavation depth (m)	Critical load ratio		
	*α*_*w*_	*α*_*s*_	*α*_*ws*_
0	1.0000	0.9992	0.9992
1	1.0000	1.0001	1.0001
2	0.9999	1.0031	1.0031
3	1.0000	1.0000	1.0000
4	1.0000	1.0003	1.0004
5	0.9999	1.0008	1.0007

### Influence of excavation depth

**Figure 7:**
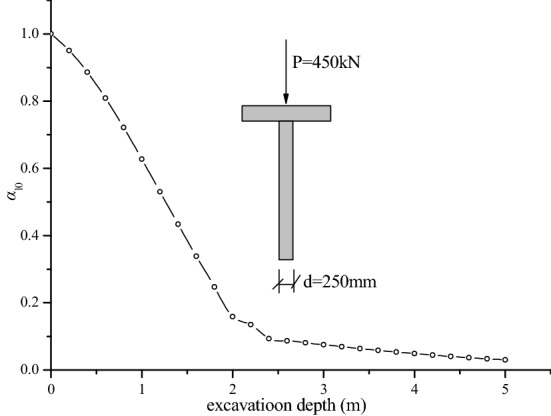
Impact of excavation depth on the critical load.

In the basement excavation beneath the existing buildings, with the increasing excavation depth, the load transfer mechanism of underpinning piles will also change. Therefore, the excavation depth is a parameter that needs to be controlled in the excavation project. In Fig. 7, *α*_l0_ is the ratio between the critical load of foundation piles at excavation depth *l*_0_ and that before excavation. Figure 7 shows that with the increasing excavation depth, the critical load ratio of underpinning piles decreased sharply. When the excavation depth reached 5 m, the ratio decreased to 0.03, and the critical load decreased by 33.3 times. The explanation may be that with the increasing excavation depth, the soil layer constraint around the pile on the foundation pile gradually disappears, resulting in the free length increase of the foundation pile, which is likely to cause buckling and instability of foundation piles.

### Influence of pile diameter

**Figure 8:**
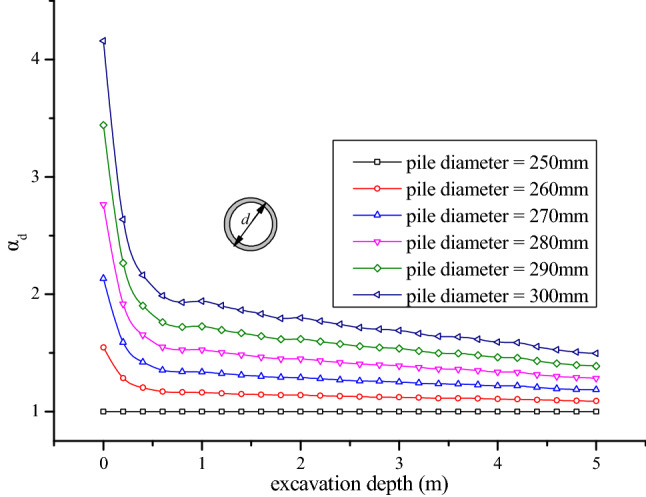
Influence of pile diameter on the critical load at different excavation depths.

In this project, steel pipe piles with diameters of 250 mm and 300 mm were used for foundation underpinning. Therefore, the influence of pile diameter of underpinning piles on the critical buckling load would be analyzed in this section. In Fig. 8, *α*_*d*_ is the ratio between the foundation pile’s critical load with diameter *d* and that with diameter 250 mm at the same excavation depth. Figure 8 illustrates that at the same excavation depth, the critical load ratio of different pile diameters (*α*_*d*_) also gradually increased with the increase of pile diameters. For instance, when the excavation depth was 1 m, the critical load ratio with pile diameters 300 mm *α*_300_ was 1.94. When the pile diameter changed from 250 to 300 mm, the corresponding critical load on the pile top increased by 94%. The reason may be that, with the augment of pile diameter, both the pile’s flexural strain energy and the pile side soil’s elastic deformation energy increase correspondingly, so that the foundation pile’s critical load also increases gradually.

## Conclusions

Based on the Ganshuixiang project, this paper explored the impact of basement excavation beneath existing buildings on the underpinning pile’s buckling stability through theoretical analysis. The following conclusions could be obtained:

1. According to the Winkler elastic foundation beam theory, the pile-soil system’s total potential energy equation was constructed in this paper. By using the minimum potential energy theory, the expression of the critical buckling load and calculation length of underpinning piles during basement excavation beneath existing buildings was obtained.
2. The influencing factors of the critical buckling load of underpinning piles during basement excavation beneath existing buildings were analyzed. It was found that the underpinning pile’s critical load converged with the half-wave number increase. In this paper, half-wave number *n* = 15. The pile skin friction and deadweight had a negligible influence on the underpinning pile’s critical load. In addition, the critical load of underpinning piles decreased sharply with the increasing excavation depth and gradually increased with the augment of pile diameter.
